# Modified Nuss procedure with a novel steel bar in patients with pectus excavatum post-congenital heart surgery

**DOI:** 10.1093/icvts/ivab284

**Published:** 2021-10-18

**Authors:** Siming Liu, Lei Wang, Hongkun Zhang, Wenhui Zeng, Fengqing Hu, Haibo Xiao, Guoqing Li, Ju Mei, Jiaquan Zhu

**Affiliations:** Department of Cardiothoracic Surgery, Xinhua Hospital, Shanghai Jiaotong University School of Medicine, Shanghai, China

**Keywords:** Pectus excavatum, Congenital cardiac surgery, Nuss procedure, Minimally invasive surgery

## Abstract

**OBJECTIVES:**

Pectus excavatum (PE) can be secondary in patients who underwent sternotomy for cardiac surgery. Retrosternal adhesions increase the complexity and risk of traditional Nuss repair. Thus, we summarized the outcomes of our modified Nuss procedure using a newly designed bar.

**METHODS:**

A retrospective analysis was performed on 35 patients who underwent modified PE repair after open heart surgery from January 2011 to July 2019. The surgery was performed using a novel bar with no need for intraoperative reshaping and rotation, assisted by thoracoscopy and subxiphoid incision when necessary.

**RESULTS:**

There were 19 males and 16 females with a median age of 5.3 years (interquartile range, 4.1–10.9) at PE repair. All patients underwent the modified procedure uneventfully with no death. The median operating time was 70 min. Twenty-nine (82.9%) patients required subxiphoid incision assistance. There was 1 case (2.8%) with unexpected sternotomy due to intraoperative bleeding. The median length of postoperative hospital stay was 4 days. During the median 3.5 years of follow-up, no bar dislocation was found and 30 (85.7%) patients had their bars removed with no recurrence recorded. After PE repair, the Haller index improved significantly (2.6 ± 0.4 vs 4.9 ± 1.3, *P* < 0.05) and further decreased till the time of bar removal (2.5 ± 0.4 vs 2.6 ± 0.4, *P* < 0.05). All patients were satisfied with the cosmetic outcome.

**CONCLUSIONS:**

The novel bar can be placed and removed easily with a low rate of adverse events. This modified Nuss procedure seems to be a safe, effective and convenient approach for the management of PE after cardiac surgery.

**Figure:**
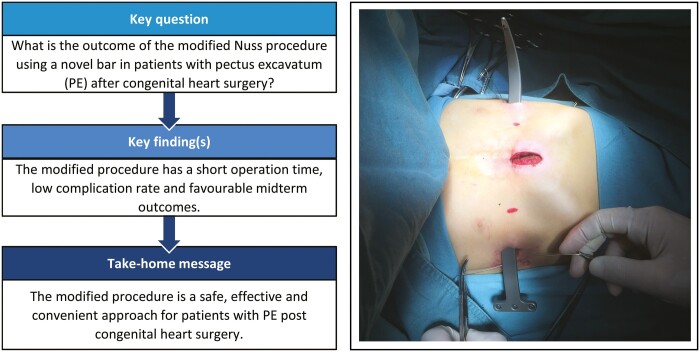

## INTRODUCTION

Pectus excavatum (PE), as the most common thoracic deformity, can develop after sternotomy for congenital heart disease. Sternal mechanical compression and severe substernal adhesions can result in cardiopulmonary dysfunction and increase the complexity of subsequent repairs, as well as the risk of adverse events in these patients [1].

Nowadays, as a milestone surgical procedure, Nuss repair has treated numerous PE patients for long-lasting and better cosmetic outcomes [2]. Nevertheless, for PE patients with severe complex deformities or with a history of thoracic surgery, the unsatisfactory outcome occurs more often, and adverse events have a higher probability than that of straightforward patients, with the most serious risk being intraoperative heart injury [3]. There have been many modifications of the Nuss repair in the last decades, including sternum elevation and thoracoscope assistance [2, 4]. However, the risk of heart injury was still as high as 7% during bar placement and 2% during bar removal [5].

In 2008, we modified the Nuss procedure with a novel bar that did not need intraoperative reshaping and rotation. According to the Nuss criteria, our early experiences had achieved favourable results in thousands of PE patients [6, 7]. The purpose of the study was to summarize the outcomes and prognosis of the new bar in PE patients after congenital heart surgery.

## METHODS

### Ethical statement

The study was approved by the Xinhua Hospital Institutional Review Board and the consent from patients was waived (Approval No. XHEC-D-2020-177).

### Study design

A retrospective screening was performed on all the patients who had PE repair in our hospital from January 2011 to July 2019. Those who had PE repair with a history of cardiac surgery and underwent the modified Nuss procedure were included. Patients who had a history of interventional cardiac surgery, concurrent primary cardiac surgery and PE repair or non-cardiac surgical sternotomy, such as Ravitch repair, were excluded. All the patients had 2 or more of the Nuss surgical indications [8]. The medical records were reviewed for demographics, disease history, perioperative imaging, operative data, postoperative complications and outcomes.

According to Park’s criteria, the morphology of deformity was classified into symmetric and asymmetric (including eccentric and unbalanced) types [9]. The Haller index (HI) was calculated using the chest computerized tomography (CT) scan data. The deformity could be divided into 3 degrees of severity according to the HI value: mild (2.50 < HI ≤ 3.25), moderate (3.25 < HI ≤ 3.50) and severe (HI > 3.50) [10]. Regarding the postoperative results, we reached a consensus using the Nuss criteria as follows [11]: excellent, if preoperative symptoms were resolved and the chest appearance was normal; good, if preoperative symptoms were resolved and the chest appearance was improved; fair, if preoperative symptoms were improved but the appearance was not completely normal; and failed, if preoperative symptoms were not improved and the chest appearance was not completely normal or deformity reoccurred.

### Operative technique and management

Based on the traditional minimally invasive Nuss repair, the modified procedure applied self-designed novel bars with right unilateral thoracoscopy [7], assisted with a subxiphoid small incision when necessary.

#### The novel steel bar

**Figure 1:**
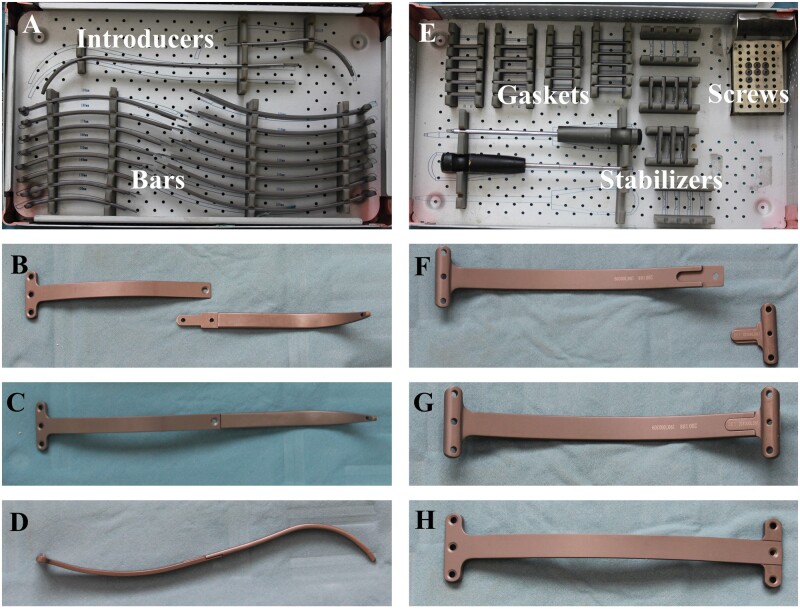
Bars and accessories. (**A**) Bars of 15 sizes and introducers of 2 sizes; (**B**) bar and corresponding introducer (disconnected); (**C** and **D**) anterior and lateral views of the connected composite of bar and introducer; (**E**) stabilizers, gaskets and screws; (**F**) bar and stabilizer (disconnected); and (**G** and **H**) posterior and anterior views of the composite of bar and stabilizer.

A set of novel steel bars (Shanghai Puwei Medical Instrument Co., Ltd, Shanghai, China) consists of bars in 15 sizes varying from 12 to 26 cm, bent from production with no need for intraoperative reshaping. Bar accessories include the introducer, stabilizer and gasket. One bar end was designed as a T-shaped stabilizer, and the other end can be connected to an introducer or a stabilizer. The gaskets can increase the length or thickness of the stabilizer when treating unbalanced deformity or facing a vast intercostal space. Titanium bars were routinely prepared for patients allergic to stainless steel (Fig. 1).

**Figure 2:**
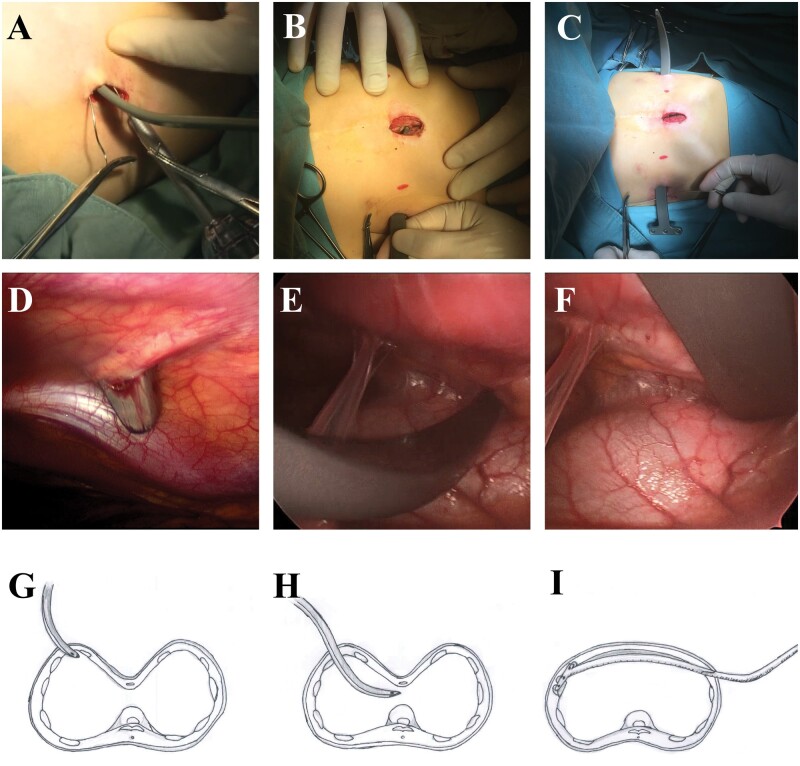
Steps of bar placement. (**A**) Puncture of the introducer tip into the right chest; (**B**) the tip of the introducer went through the retro-sternal adhesion at the lowest point; (**C**) the introducer came out from the left chest wall, and the bar was pulled to the expected position with no need for rotation, enabling pectus excavatum improvement; (**D**–**F**) intrathoracic views of (A)–(C); and (**G**–**I**) schematics of (A)–(C).

The modified Nuss procedure was performed by a team of both cardiac and thoracic surgeons. The main steps are as follows:

1. Right-side thoracoscopy was routinely used to provide direct visualization when dissecting adhesions and crossing the bar.
2. Through a 2-cm subxiphoid incision, the retrosternal adhesion was dissected by index finger and cautery. The thoracoscope could be inserted into this incision to provide clearer vision when necessary.
3. Bilateral 2-cm vertical incisions were made at the cross points of the deepest deformity and anterior-axillary line. From this incision to the highest point of the pectus ridge, subcutaneous tunnels were made for bar crossing. Then, a bar of appropriate size was connected to the introducer, entered and exited the space beneath the valley of deformity (Fig. 2A, B, D, E, G and H). As the bar advanced towards the left vertical incision, the deformity was corrected by pulling up the bar and pushing down the chest wall (Fig. 2C, F and I). Then, the stabilizer took the place of the introducer, and the composite bar was secured to the bilateral ribs with steel wires.
4. A drainage tube was occasionally placed to prevent pneumothorax or pleural effusion after dissecting a severe adhesion. Before closing the thoracic cavity, positive end-expiratory pressure ventilation was applied to inflate lungs and expel air.

Intravenous analgesia pumps were used for 2–3 days after the operation, followed by oral medication over 1 week. All patients received intravenous antibiotics for 2 days after the surgery. Patients were advised to avoid any physical activities for 6 weeks and gradually resume activities and sports up to 3 months. The bar should stay *in situ* for at least 2 years, as long as the rib cage has average growth.

### Follow-up and bar removal

Postoperative follow-up was performed periodically and consisted of physical examination, chest X-ray (or CT scan) and echocardiogram. Usually, after 2 years of the PE repair, the patients were recruited to remove the implanted steel bars. A pre-removal CT scan was routinely carried out for assessment. The bar removal operation was performed under general anaesthesia with a laryngeal mask airway. The previous incisions were opened, and the composite bars and wires were dissected and removed. A postoperative chest X-ray was performed to ensure the complete removal of the implanted devices. From then on, follow-up was conducted once a year.

### Statistical analysis

Continuous data are expressed as mean ± standard deviation or the median and interquartile range (IQR) depending on the data distribution, as assessed using the Shapiro–Wilk test. Analysis of HI and thoracic diameters measured at different time points was performed with one-way repeated-measures analysis of variance, and the comparisons within the groups were performed with Bonferroni test. STATA 14.0 software (STATA Corporation, College Station, TX, USA) was used for statistical analyses. A *P*-value of <0.05 was considered statistically significant.

## RESULTS

### Demographics

A total of 35 patients who underwent the modified Nuss procedure with a history of cardiac surgery were reviewed in the present study and accounted for 2.0% of all PE repair patients during the same period. There were 19 males and 16 females, with a median age of 5.3 years (IQR, 4.1–10.9). The main clinical manifestations included chest tightness in 7 patients and growth retardation in 2 patients, and the remaining patients sought medical attention for sternal deformity. Thirty-one patients (88.6%) had symmetric type PE, and the other 4 had asymmetric PE. The median preoperative HI was 4.9 (IQR, 3.9–5.8, *n* = 35), and 33 patients (94.3%) had a severe degree of deformity.

History of cardiac disease is shown in Table 1. Ventricular septal defect (48.6%) and atrial septal defect (25.7%) were the 2 most common congenital heart diseases. No residual cardiac abnormalities existed, except for 1 patient who had unilateral pulmonary vein stenosis after previous total anomalous pulmonary venous connection surgery. The median age at primary cardiac surgery was 1.0 years (IQR, 0.5–3.8). Concerning the onset of thoracic deformity, 31 cases (88.6%) had compression post heart surgery, and in 71% of them (22/31), this occurred within 1 year. The median interval between primary heart surgery and PE repair was 3.9 years.

**Table 1:** Preoperative characteristics of the patients

Characteristics	Number of patients (%)/median (IQR)
Gender	
Male	19 (54.3)
Female	16 (45.7)
Age at PE repair (years)	5.3 (4.1–10.9)
Classification^a^	
Symmetric	31 (88.6)
Eccentric	3 (8.6)
Unbalanced	1 (2.8)
Degree of deformity	
Severe (HI > 3.50)	33 (94.3)
Moderate (3.50 ≥ HI > 3.25)	2 (5.7)
Mild (3.25 ≥ HI > 2.50)	0 (0)
History of CHD^a^	
Ventricular septal defect	17 (48.6)
Atrial septal defect	9 (25.7)
AVSD	2 (5.7)
Patent ductus arteriosus	4 (11.4)
Patent foramen ovale	4 (11.4)
Tetralogy of Fallot	3 (8.6)
Pulmonary venous drainage	4 (11.4)
Aortic stenosis	1 (2.8)
Pulmonary artery stenosis	1 (2.8)
Mitral regurgitation	1 (2.8)
Onset of PE	
Before cardiac surgery	4 (11.4)
After cardiac surgery	31 (88.6)
Age at prior cardiac surgery (years)	1.0 (0.5–3.8)
Interval between cardiac surgery and PE repair (years)	3.9 (2.5–5.2)

AVSD: atrioventricular septal defect; CHD: congenital heart disease; HI: Haller index; IQR: interquartile range; PE: pectus excavatum.

a Some patients combined multiple heart diseases.

### Operative data of modified Nuss procedure

All the patients completed the operation uneventfully. The median operating time was 70 min (IQR, 55–107), and the intraoperative blood loss was 10 ml (IQR, 5–20). Twenty-nine patients (82.9%) had subxiphoid incisions assistance, and in 13 (37.1%) patients, a unilateral chest tube was placed or mediastinal drainage took place, with a median volume of 200 ml. The majority of patients (94.3%) achieved the desired correction effect with 1 bar. Two cases in this study needed the placement of 2 bars due to the broad-flat deformity. The median length of postoperative hospital stay was 4.0 days (IQR, 4–6) (Table 2).

**Table 2:** Perioperative data of the modified Nuss procedure

Variable	Number of patients (%)/median (IQR)
Operating time (min)	70 (55–107)
Intraoperative blood loss (ml)	10 (5–20)
Subxiphoid incision	29 (82.9)
Drainage tube placement	13 (37.1)
Drainage volume (ml)	200 (74–526)
Number of bars	
One	33 (94.3)
Two	2 (5.7)
Complications	
Pleural effusion	2 (5.7)
Pneumonia	2 (5.7)
Severe infection	1 (2.8)
Cardiac injury	1 (2.8)
Length of hospital stay (days)	4 (4–6)
Follow-up period (years)	3.5 (2.5–5.4)

IQR: interquartile range.

Moreover, 3 patients underwent full sternotomy during PE repair. Two of them were scheduled. One needed concurrent reoperation for pulmonary vein stenosis after prior total anomalous pulmonary venous connection surgery, with the operating time being 261 min. The other was due to sternal pseudo-arthrosis and needed re-wiring of the whole sternum before bar placing. The only unscheduled sternotomy occurred in a 15-year-old boy who suffered right atrial bleeding while we dissected a dense adhesion between the right atrium and the sternum. The rupture was repaired immediately by a cardiac surgeon under cardiopulmonary bypass, and then, a bar was placed by a thoracic surgeon. This operation took 295 min. The patient recovered well and was discharged 8 days post the operation. Heart injury occurred only once as explained above and has never occurred again since 2012.

Complications included pneumonia in 2 patients (5.7%) and pleural effusion in 2 patients (5.7%) with no need for drainage. None of the patients developed haemothorax, pneumothorax, wound haematomas, bar displacement or death.

### Improvement in the thoracic cage

**Figure 3:**
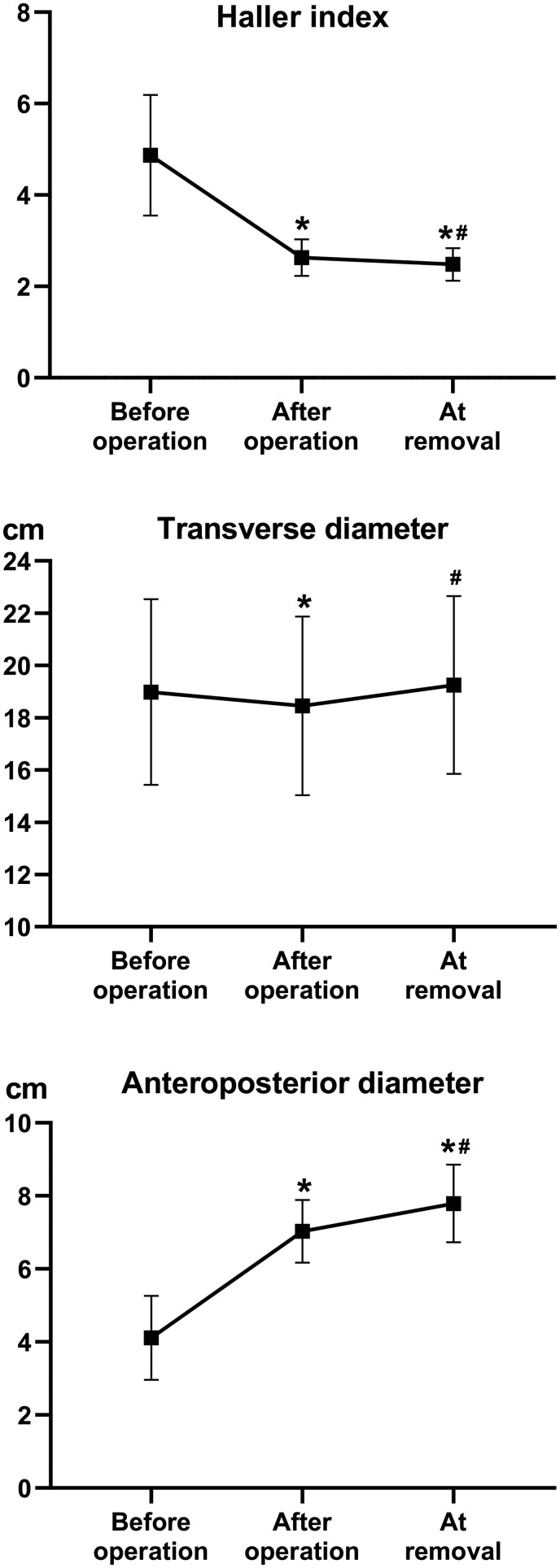
Comparison of image data measurement at 3 time points (*n* = 27 pairs). Haller index, anteroposterior diameters and transverse diameters were significantly improved after operation and this improved in the follow-up years. * versus before operation group, *P* < 0.05; # versus after operation group, *P* < 0.05.

Reviewing the patients who had bar removed, 27 patients had complete imaging data at the 3 time points: before PE repair (T1), immediately after PE repair (T2) and at bar removal (T3). Comparisons of HI value, transverse diameters (TD) and anteroposterior diameters (APD) at the 3 time points are in Fig. 3. The HI, TD and APD changed significantly within the 3 time points. Subsequent pairwise comparisons of HI indicated significant differences (T1 = 4.9 ± 1.3; T2 = 2.6 ± 0.4; T3 = 2.5 ± 0.4; all *P* < 0.05). Pairwise comparisons of TD between T1 and T2 and between T2 and T3 revealed significant differences (both *P* < 0.05), but this was not evident between T1 and T3 (*P* = 0.22) (T1 = 19.0 ± 3.5 cm; T2 = 18.5 ± 3.4 cm; T3 = 19.3 ± 3.4 cm). All the pairwise comparisons of APD among the 3 time points reached a significant level (T1 = 4.1 ± 1.1 cm; T2 = 7.0 ± 0.9 cm; T3 = 7.8 ± 1.1 cm; all *P* < 0.05).

### Bar removal operation and follow-up

During the median follow-up period of 3.5 years (IQR, 2.5–5.4), a total of 30 patients had their bars removed, with the average time for bar staying in being 25.4 ± 4.7 months. In these patients, the mean operating time of bar removal was 36 ± 13 min and the estimated blood loss was 5.0 ± 3.2 ml. No significant complication associated with bar removal was observed. No recurrence was recorded till now.

After PE repair, the initial observation revealed that 33 patients (94.3%) had excellent results and 2 patients (5.7%) had good results according to the Nuss criteria. None of the patients had a fair or failed outcome. As 30 patients had their bars removed, 28 (93.3%) showed excellent outcomes and the remaining 2 (6.7%) had good results. After 2 years of bar removal, the midterm results of 18 available patients included excellent results in 17 (94.4%), good results in 1 (5.6%) and no poor results.

## DISCUSSION

Generally, for patients undergoing Nuss repair after sternotomy, the adhesions between the sternum and pericardium (or heart) greatly increase the risk of the operation and the incidence of adverse events [2]. Therefore, adequate dissection of adhesions is critical for heart protection in these patients.

Based on the traditional Nuss bar, we have optimized the bar itself and the surgical technique and obtained an excellent surgical experience [7]. As the most distinctive feature, the new bar has a fixed bend and does not need rotation during the operation. The bar had been bent upon manufacture, and it simulated the human standard anterior-thoracic shape. A non-rotation procedure, as described before, does not require aggressive substernal dissection as does the traditional procedure and thus leads to less trauma and a shorter operation time. Furthermore, by using T-shaped stabilizers, it is more stable as the supporting force is located on the bilateral ribs instead of the intercostal muscles.

Adequate vision and tactile feedback played an essential role during the dissection of retrosternal adhesions. Thoracoscopy, commonly used in minimally invasive thoracic surgery, can provide a direct vision for delicate surgical procedures [2]. A small subxiphoid incision can provide a pathway for both sharp and blunt separations. When performing blunt dissection with the finger, tactile feedback played an essential role in preventing heart damage [12]. Sternal elevation can at times facilitate adhesion separation and bar crossing for patients with severe deformity and adhesions [13, 14]. Meanwhile, since excessive dissection may lead to an apparent inflammatory response, it is reasonable to place chest tubes helping patients to get a better recovery [15].

PE can be secondary after sternotomy but has not been well reported due to its rare incidence [16]. In this study, 5 cases underwent previous cardiac surgeries in our hospital, accounting for 0.17% (5/3012) of total patients who had sternotomy for congenital heart surgery (<16 years old) during the same period. Concerning the onset of the deformity, 31 patients (88.6%) had secondary PE. The proportion of symmetrical deformity in them was as high as 90.3%, and deformity often occurred within 1 year of cardiac surgery (71%), which is significantly different from the primary. Some studies showed the feasibility of concurrent surgery for congenital heart disease and PE [17, 18]. However, we prefer to correct the cardiac disease first, especially in those under 3 years old, unless the surgery can be completed by minimally invasive incision or intervention.

After PE repair, the shape of the thorax was apparently corrected and all the measurement showed improvement. Comparing the 27 cases where imaging CT scan data was available, the HI was significantly decreased after repair and the value further decreased after bar placement. The APD increased apparently after the operation and continued to increase during the follow-up time. The TD decreased immediately after the bar placement due to the thoracic shape turning to a round circle, and it gradually increased as the children grew, which resulted in no significant difference between T1 and T3.

As mentioned before, only one case of heart injury happened in our centre, and its incidence was 2.9%, minor than that reported by Chen *et al.* [13] and Jaroszewski *et al.* [5], with a rate of 6.1% and 7%, respectively. Presently, we cooperate with cardiac surgeons to dissect the substernal adhesions to minimize the risk of heart injury among this high-risk population, with emergent cardiopulmonary bypass availability. No heart injury occurred thereafter.

This study is limited by its retrospective nature with few cases. Only short- and midterm follow-up data are available, and we are looking forward to the long-term outcomes. A propensity score matching study may be performed to find the difference in this high-risk population; however, we did not conduct this due to the relatively good outcomes of our experiences.

## CONCLUSION

The modified Nuss procedure using the novel bar is safe, effective and convenient in patients with PE after congenital cardiac surgery, as it retains the growth of the thoracic cage. Increased visualization with thoracoscopy assistance and the subxiphoid incision may potentially reduce heart injuries during the surgery. Our strategy provides an alternative method in high-risk patients with previous cardiothoracic surgery.

## Abbreviations

**ABBREVIATIONS **
APD: Anteroposterior diameters
HI: Haller index
IQR: Interquartile range
PE: Pectus excavatum
TD: Transverse diameters
